# Experimental Investigation of Radiation Shielding Competence of Bi_2_O_3_-CaO-K_2_O-Na_2_O-P_2_O_5_ Glass Systems

**DOI:** 10.3390/ma14175061

**Published:** 2021-09-03

**Authors:** Dalal Abdullah Aloraini, Aljawhara H. Almuqrin, M. I. Sayyed, Hanan Al-Ghamdi, Ashok Kumar, M. Elsafi

**Affiliations:** 1 Department of Physics, College of Science, Princess Nourah Bint Abdulrahman University, Riyadh 11671, Saudi Arabia; daalorainy@pnu.edu.sa (D.A.A.); ahalmoqren@pnu.edu.sa (A.H.A.); hmalghmdi@pnu.edu.sa (H.A.-G.); 2 Department of Physics, Faculty of Science, Isra University, Amman 11622, Jordan; 3 Department of Nuclear Medicine Research, Institute for Research and Medical Consultations (IRMC), Imam Abdulrahman Bin Faisal University (IAU), P.O. Box 1982, Dammam 31441, Saudi Arabia; 4 Department of Physics, University College, Benra, Dhuri 148024, India; ajindal9999@gmail.com; 5 Department of Physics, Punjabi University, Patiala 147002, India; 6 Physics Department, Faculty of Science, Alexandria University, Alexandria 21511, Egypt; mohamedelsafi68@gmail.com

**Keywords:** radiation, glasses, mass attenuation coefficient, NaI (Tl) scintillation detector

## Abstract

The gamma-ray shielding features of Bi_2_O_3_-CaO-K_2_O-Na_2_O-P_2_O_5_ glass systems were experimentally reported. The mass attenuation coefficient (MAC) for the fabricated glasses was experimentally measured at seven energy values (between 0.0595 and 1.33 MeV). The compatibility between the practical and theoretical results shows the accuracy of the results obtained in the laboratory for determining the MAC of the prepared samples. The mass and linear attenuation coefficients (MACs) increase with the addition of Bi_2_O_3_ and A4 glass possesses the highest MAC and LAC. A downward trend in the linear attenuation coefficient (LAC) with increasing the energy from 0.0595 to 1.33 MeV is found. The highest LAC is found at 1.33 MeV (in the range of 0.092–0.143 cm^−1^). The effective atomic number (Z_eff_) follows the order B1 > A1 > A2 > A3 > A4. This order emphasizes that increasing the content of Bi_2_O_3_ has a positive effect on the photon shielding proficiencies owing to the higher density of Bi_2_O_3_ compared with Na_2_O. The half value layer (HVL) is also determined and the HVL for the tested glasses is computed between 0.106 and 0.958 cm at 0.0595 MeV. The glass with 10 mol% of Bi_2_O_3_ has lower HVL than the glasses with 0, 2.5, 5, and 7.5 mol% of Bi_2_O_3_. So, the A4 glass needs a smaller thickness than the other glasses to shield the same radiation. As a result of the reported shielding parameters, inserting B_2_O_3_ provides lower values of these three parameters, which in turn leads to the development of superior photons shields.

## 1. Introduction

Nuclear medicine and radiation shielding technology provide flexible tools that play an outstanding role in current human life. Radiation technology have contributed to industrial or economic growth such as dental or medical applications (CT, X-ray, PET); pasteurize foods (radiations are used to kill bacteria); neutron activation analysis (radiations are used to find the material composition); and gamma sources such as ^60^Co, ^192^Ir, or X-rays, which are used to check the weld defects in industries, communication system, nuclear reactors, material science, and student research facilities [1]. On the other hand, these radiations have dangerous effects after interactions with living tissue. Radiations also cause burn in a very short time and increase the risk of cancer. To diminish the intensity of the radiations to a safe level, a superior grade of protective materials is required. Radiation protection is based on three factors, that is, shielding, distance, and time [2,3]. Lead, clay bricks, tiles, and concrete and other traditional materials were used as shielding material for many years to make the environment safe and protect workers from hazardous radiations [4,5]. On the other hand, particular types of glass systems have been made for windows, isotope containers, and X-ray imaging systems [6]. Glass systems mainly comprise modifier, former, and intermediate oxides. The addition of heavy metal oxide like bismuth oxide (Bi_2_O_3_) in forming cations like P^5+^, B^3+^, and Si^4+^ may form glasses. Bi^3+^ ions have high polarizability, which prevents melt crystallization through asymmetry, which comes from oxygen polyhedral. Bismuthate glasses mainly consist of BiO_6_ octahedral units and BiO_3_ pyramidal [7].

Many researchers reported shielding properties like mass attenuation coefficient of various heavy metal oxide-doped silicates and borates glasses. Kirdsiri et el. reported the radiation shielding properties of BaO-, PbO-, and Bi_2_O_3_-doped silicate glasses at 662 keV gamma ray energy; Limkitjaroenporn et al. prepared lead sodium borate glasses and studied the shielding, structural, and optical properties of these glasses; and Al-Hadeethi et el. studied the shielding properties of germanate lead calcium alumina glass system, among others [8,9,10]. However, the reports of phosphate glasses are very limited in radiation shielding applications. Phosphate glasses have excellent properties over other glasses including marvelous visible light transmission, low melting temperature, and high thermal expansion coefficient and refractive index, which makes them convenient in industries and shielding applications. The addition of metal oxides to the glasses upgrade the chemical, physical, and shielding features of the glasses. However, lead is toxic in nature, which makes it less compatible for shielding applications. So, bismuth is a good alternative to lead, which is used in the shielding field [11].

In our recent findings regarding the samples Bi_2_O_3_-P_2_O_5_-CaO-Na_2_O-K_2_O, we have reported the structural and the gamma ray shielding behavior between 0.015 MeV and 15 MeV [12]. In continuation of the work in the present part, the experimental studies were conducted to measure the gamma ray shielding parameters in the narrow beam setup with NaI(Tl) detector using the point sources Am-241, Ba-133, Cs-137, and Co-60. The obtained data were verified with the data obtained using XCOM.

## 2. Materials and Methods

### 2.1. Sample Preparations

**Figure 1:**
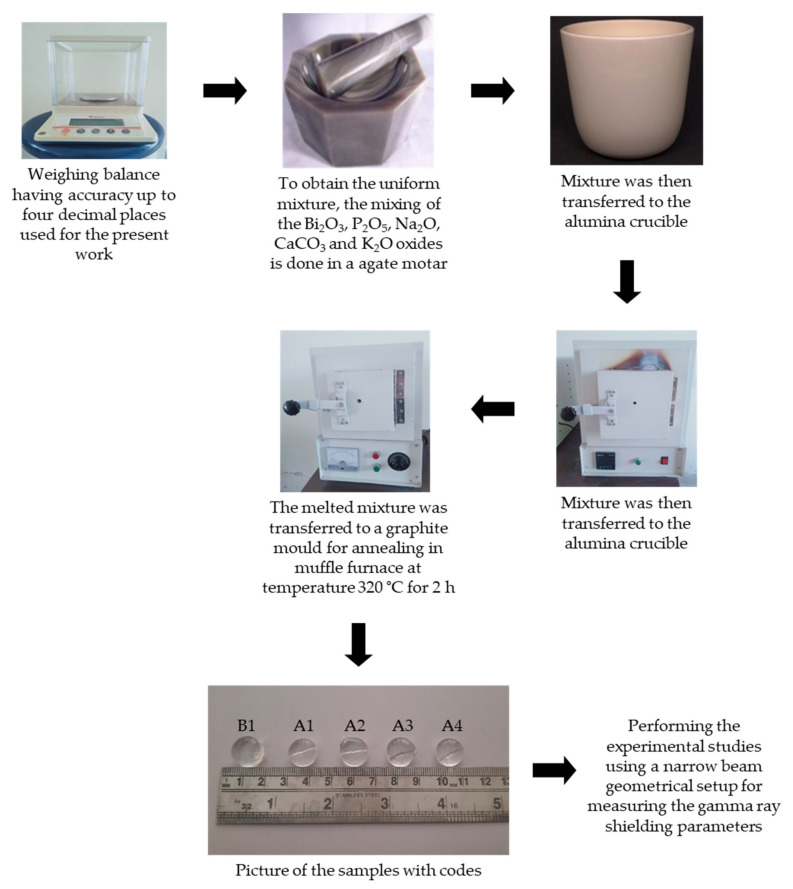
Graphical procedure for the preparation of the prepared glasses.

The details of the steps followed for the preparation and the density measurement have been discussed in our recent publication [12]. The pictorial representation for the procedure is depicted in Figure 1. The samples have been coded as follows:

B1: 20 CaO-10 K_2_O-30 Na_2_O-40 P_2_O_5_ (density = 2.3337 g/cm^3^, thickness = 0.6430 cm)

A1: 2.5 Bi_2_O_3_-20 CaO-10 K_2_O-27.5 Na_2_O-40 P_2_O_5_ (density = 2.8605 g/cm^3^, thickness = 0.5736 cm)

A2: 5 Bi_2_O_3_-20 CaO-10 K_2_O-25 Na_2_O-40 P_2_O_5_ (density = 3.0709 g/cm^3^, thickness = 0.6570 cm)

A3: 7.5 Bi_2_O_3_-20 CaO-10 K_2_O-22.5 Na_2_O-40 P_2_O_5_ (density = 3.2884 g/cm^3^, thickness = 0.6904 cm)

A4: 10 Bi_2_O_3_-20 CaO-10 K_2_O-20 Na_2_O-40 P_2_O_5_ (density = 3.5182 g/cm^3^, thickness = 0.5516 cm)

### 2.2. Measurement of Gamma Ray Shielding Parameters

The LAC was experimentally calculated using a NaI (Tl) scintillation detector and different radioactive point sources. The detector was shielded and connected to the Win-TMC software and the background radiation was examined and placed as a subtracted file for other main measurements. The point sources Am-241, Ba-133, Cs-137, and Co-60 were used in the measurements to cover a broad range of energy. The initial activity of these sources on 1 June 2009 was 259 kBq, 275.3 kBq, 385 kBq, and 212.2 kBq, respectively.

**Figure 2:**
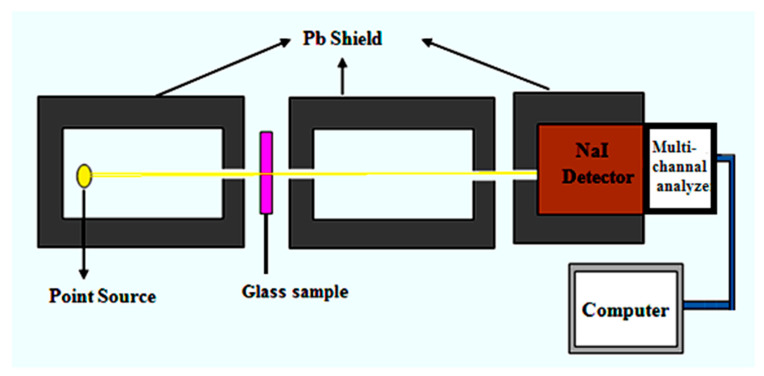
Experimental set-up.

The detector energy was calibrated using Cs-137 and Co-60 sources [13,14,15]. The collimated beam method was used and the sample was placed between the source and the detector as shown in Figure 2. After sufficient run time, the count rate for each peak (line) can be obtained directly from the screen of the computer. The count rate represents the intensity of the beam, so the initial intensity (count rate without sample) and transmitted intensity (count rate within sample) can be calculated. The LAC can be estimated from Equation (1) [16]:

(1) $$\mathrm{LAC}=\frac{1}{x}\ln\left(\frac{I_{0}}{I}\right)$$

The MAC is an important parameter and it is not affected by the density of an absorber, and the measured value was obtained using the following Equation (2) [17].

MAC = LAC/ρ(2)

where ρ is the density of the measured sample. The MAC was calculated theoretically using XCOM software and compared with the results of experimental data. The HVL is the thickness needed to reduce the intensity of the incoming photons by 50% and its Equation (3) as follows [18]:

(3) $$\mathrm{HVL}=\frac{\ln2}{\mathrm{LAC}}$$

The mean free path (MFP) is described by the following equation [18]:

(4) $$\mathrm{MFP}=\frac{1}{\mathrm{LAC}}$$

The effective atomic number (Z_eff_) for the studied glass can be obtained using Equation (5) [19]:

(5) $$Z_{\mathrm{eff}}=\frac{\sum_{i}f_{i}A_{i}{\left(\frac{\mu }{\rho }\right)}_{i}}{\sum_{j}\frac{A_{j}}{Z_{j}}{\left(\frac{\mu }{\rho }\right)}_{j}}$$

where f_i_ and A_i_, refer to the molar fraction and atomic weight of the ith constituent element in the selected glass, respectively. Moreover, we calculated the heaviness (H%) for the selected samples using Equation (6) [20]:

(6) $$\mathrm{Heaviness}\;\left(H\%\right)=\frac{\mathrm{Density}\;\mathrm{of}\;\mathrm{Composite}\;\mathrm{material}}{\mathrm{Density}\;\mathrm{of}\;\mathrm{lead}}$$

The shielding efficiency of an absorber sample can be investigated using a parameter called the radiation protection efficiency (RPE) and given by the next Equation (7) [21].

(7) $$\mathrm{RPE}=\left(1-\frac{I}{I_{0}}\right)\times 100$$

## 3. Results and Discussion

The MAC and LAC for the fabricated glasses were experimentally measured at seven energy values (between 0.0595 and 1.33 keV). To assess the validity of the experimental results, we calculated the theoretical MAC again using a XCOM program. Table 1 summarizes the (MAC)_E_ and (MAC)_T_, where E and T denote the experimental and theoretical data, respectively. Evidently, the (MAC)_E_ and (MAC)_T_ are close together, with a small deviation reported (less than 4%). As an example, we found the (MAC)_E_ for B1 at 0.081 MeV equals 0.209 cm^2^/g, close to 0.123, which was obtained by XCOM. Moreover, for A3, the measured MAC at 0.161 MeV is 0.531 cm^2^/g, which agrees with the theoretical result (i.e., 0.540 cm^2^/g), with a small difference of 1.66%. The relative deviation can be calculated by the following equation:

(8) $$\Delta \;\left(\%\right)=\left|\frac{{\left(\mathrm{MAC}\right)}_{T}-{\left(\mathrm{MAC}\right)}_{E}}{{\left(\mathrm{MAC}\right)}_{T}}\right|\times 100$$

The compatibility between the practical and theoretical results shows the accuracy of the results obtained in the laboratory for determining the MAC of the prepared samples.

**Figure 3:**
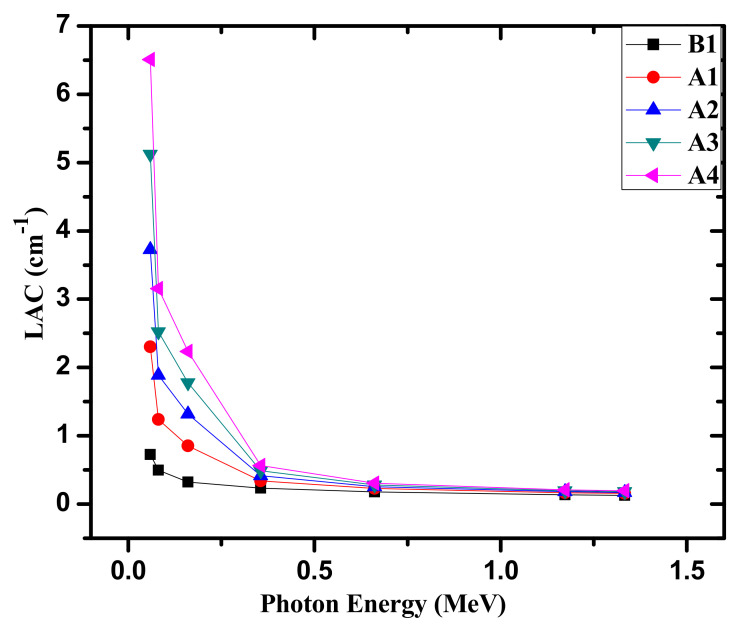
The linear attenuation coefficient for the fabricated glasses.

In Figure 3, we present the LAC for the glasses. We can easily see from the figure that the LAC increases with the addition of Bi_2_O_3_, thus at the seven examined energies, A4 possesses the highest LAC, owing to the highest amount of Bi_2_O_3_ present in this glass. Meanwhile, B1 glass, the glass with 0 mol% of Bi_2_O_3_, has the minimum LAC, and this is correct at all energies. Moreover, a reducing trend in LAC is distinguishable for the chosen glasses towards the inclining radiation energies from 0.0595 to 1.33 MeV. It is important to discuss the LAC parameter numerically in the examined energies. The results showed that the LAC changes from 0.724 to 0.497 cm^−1^ for B1 between 0.0595 and 0.081 MeV, and for the same sample changes from 0.321 to 0.232 cm^−1^ between 0.161 and 0.356 MeV. For A1, the LAC changes from 2.303 to 0.851 and to 0.114 cm^−1^ for 0.0595, 0.161, and 1.33 MeV, respectively.

**Figure 4:**
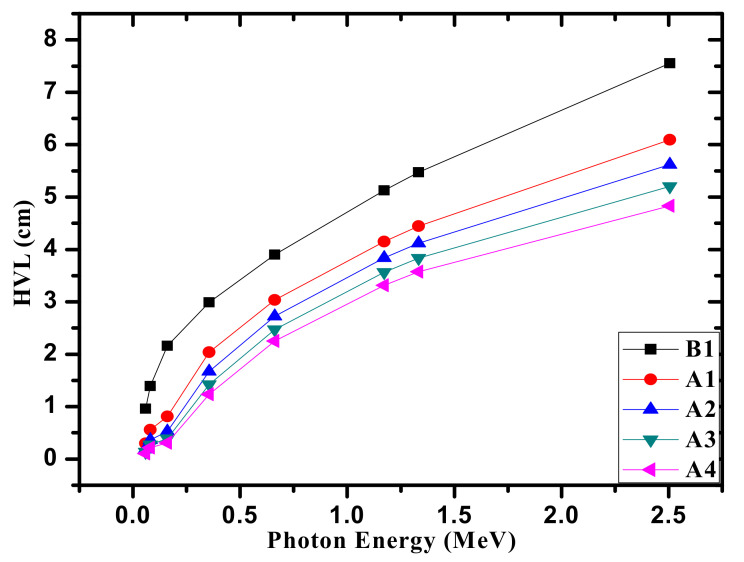
The HVL for the fabricated glasses.

Moreover, we investigated the half value layer (HVL), which can provide essential information about the radiation protection efficiency for the selected medium (the fabricated glasses in our work). We calculated the HVL for the chosen glasses at the same energies used in MAC data, namely between 0.0595 and 1.33 keV. The results are plotted and given in Figure 4. Examining the data given in this figure, one can observe easily an upward trend in HVL with increasing the energy from 0.0595 to 1.33 MeV. This result indicates that the increasing the energy of thr photon causes an increase of the ability of the photons to transmit through the sample. Additionally, from Figure 4, the smallest HVL is found at 0.0595 MeV (in the range of 0.106–0.958 cm), and a sharp increase throughout the ascending energies occurs (3.574–5.473 cm at 1.33 MeV). This situation ascertains that more photons can penetrate the chosen glasses when the energy of the radiation increases. It is to be noted from Figure 4 that inserting Bi_2_O_3_ into the glasses is an efficient way of decreasing the HVL, and thus of enhancing the attenuation capability of the chosen samples. At any energy, A4 displays a smaller HVL than that of the others. We found the HVL follows the order B1 > A1 > A2 > A3 > A4. This order emphasizes that adding more content of Bi_2_O_3_ has a positive effect on the photon shielding proficiencies owing to the higher density of Bi_2_O_3_ compared with Na_2_O. Accordingly, one can easily demonstrate that Bi_2_O_3_ can reduce the HVL, and thus A4 sample can be regarded as the best.

**Figure 5:**
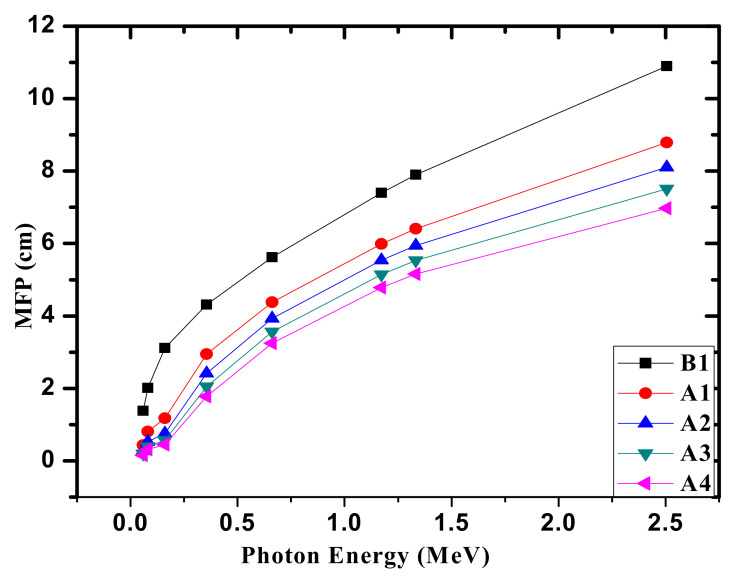
The MFP for the fabricated glasses.

Similar to the HVL, we calculated the MFP. The values of the MFP represent the inverse of the LAC. Practically, the lower the MFP, the more radiation desire shielding properties there are for the medium. In Figure 5, the relation between the MFP and the energy is presented. At all energies, the MFP have an increasing trend with the Bi_2_O_3_ content. This phenomenon can be ascribed to the increasing density of the chosen glasses from 2.3337 g/cm^3^ (for B1) to 3.5182 g/cm^3^ (for A4) thanks to the Bi_2_O_3_ insertion from 0 to 10 mol% in place of Na_2_O. At 0.0595 MeV, the increment in the amount of Bi_2_O_3_ from 0 to 10 mol% causes a reduction in the MFP values from 1.382 cm to 0.154 cm, and from 2.013 to 0.317 cm at 0.081 MeV. At higher energies (0.662 and 1.33 MeV), the MFP declines from 5.624 to 3.251 cm and from 7.896 to 5.156 cm owing to the increment in Bi_2_O_3_ from 0 to 10 mol%. Thus, we can say that A4 glass (with 10 mol% of Bi_2_O_3_) needs a smaller thickness than the other glasses to shield the same radiation. For a fixed composition, the maximum (minimum) MFP occurs at 0.0595 MeV (1.33 MeV). We can say that increasing the energy leads to an increment in the MFP.

**Figure 6:**
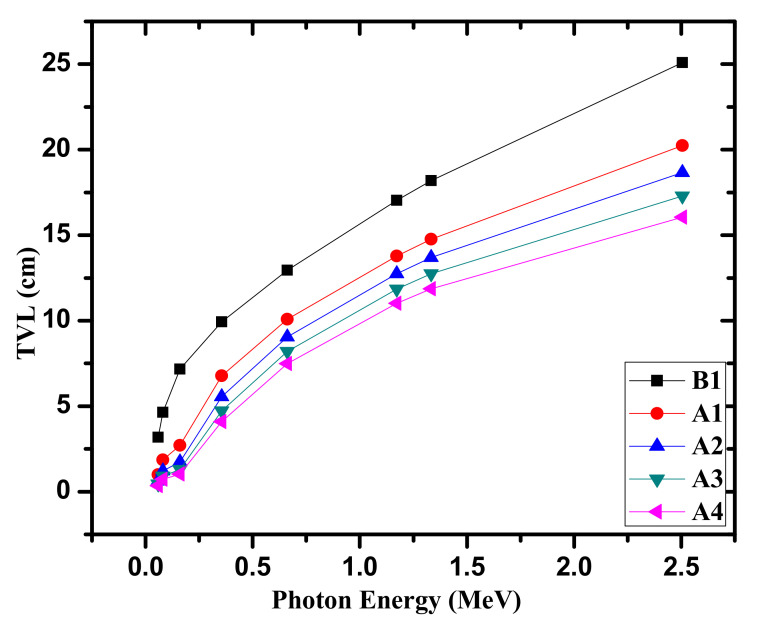
The TVL for the fabricated glasses.

We also determined the tenth value layer (TVL) for the tested B1 and A1–A4 glasses. The results of TVL as a function of the energy are exhibited in Figure 6. At the first energy (i.e., 0.0595 MeV), the TVL depends considerably on the density of the samples, because a considerable reduction in the TVL is found at this energy. The TVL values at this energy show a reducing behaviour from 3.181 cm to 0.354 cm for B1 and A4 glasses. Obviously, the TVL at other energies also depends on the density, and we can see that an increase in glass density leads to a reduction in TVL. The TVL trend reported in Figure 6 is similar to that found in the previous two figures. This is because of the fact that these parameters depend inversely on the LAC. Moreover, the TVL at 0.081 MeV takes the following values: 4.634, 1.860, 1.221, 0.913, and 0.730 cm for B1, A1, A2, A3, and A4 samples, respectively. The maximum TVL values occur at 1.33 MeV and vary between 16.052 cm for A4 and 25.098 cm for B1 glass. The high amount of B_2_O_3_ in A4 glass is responsible for the high density, and this demonstrated the low TVL of this glass. As a result of the HVL, MFP, and TVL parameters, inserting B_2_O_3_ provides lower values of these three parameters, which in turn develops superior photon shields.

**Figure 7:**
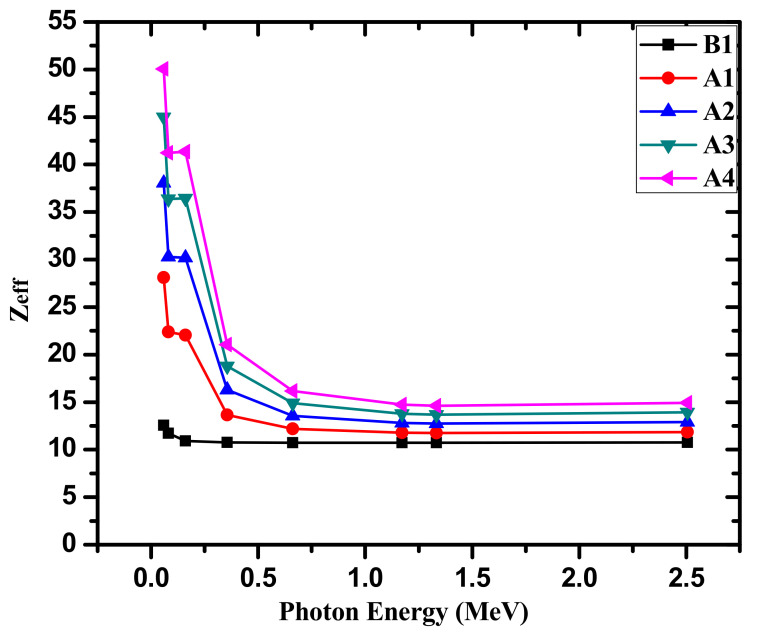
The Z_eff_ for the fabricated glasses.

We determined another essential parameter used frequently to describe the interaction between the radiation and the glasses, namely the effective atomic number (Z_eff_). Figure 7 graphs the Z_eff_ as a function of the energy for the fabricated samples. Z_eff_ depends mainly on two parameters, the energy and the chemical components of the glasses. We will discuss the dependence of these two parameters on the Z_eff_. Firstly, when we look at Figure 7, we can see that the Z_eff_ attains maximum values at 0.0595 MeV (in order of 12.55–50.06) and then decreases to 0.662 MeV, then a very slight reduction in Z_eff_ is observed between 0.662 and 1.33 MeV. As many researchers reported, the high Z_eff_ at low energy is due to the domination of the photoelectric effect [22]. Because the atomic numbers of Na and Bi are 11 and 83, it is plausible to find an increasing trend in the Z_eff_ with the replacement of Na_2_O by Bi_2_O_3_. At any energy, A4 glass predominates over the remaining glasses owing to the high Bi_2_O_3_ content (10 mol%) in BTe4. It is also found that the influence of Bi_2_O_3_ on the Z_eff_ is significant at low energy. For instance, the Z_eff_ increases from 12.55 to 50.06 at 0.0595 MeV, from 11.71 to 41.21 at 0.081 MeV, and from 10.92 to 41.43 at 0.161 MeV. Meanwhile, at higher energy (1.173 Mev, for example), Z_eff_ only increases from 10.73 to 14.75.

**Figure 8:**
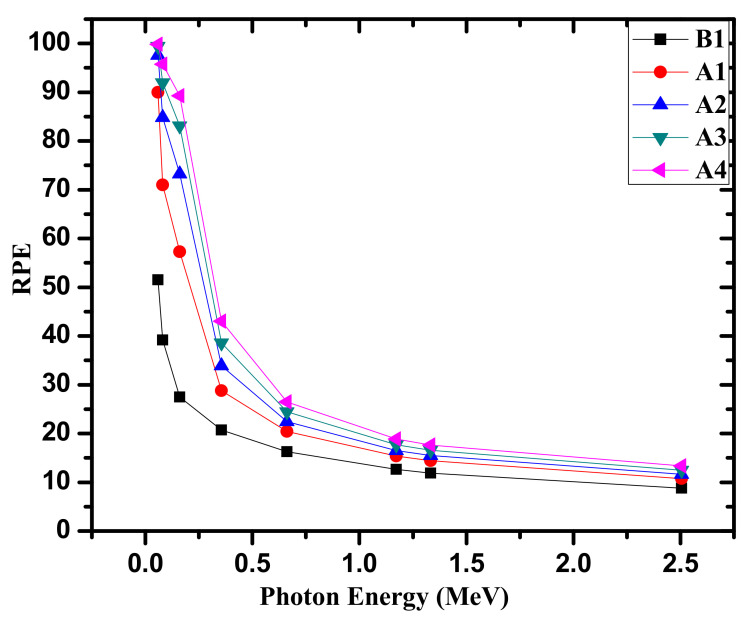
The RPE for the fabricated glasses.

To compare the shielding competence of the fabricated samples, the RPE of the present samples was plotted versus energy in Figure 8. The higher the RPE of the sample, the better its radiation shielding efficiency. Thus, it is clear from the data that the RPE improves with the increase in Bi_2_O_3_ concentration, as %RPE values are 51.51, 90.00, 97.60, 99.40, and 99.85 for the B1, A1, A2, A3, and A4 samples, respectively, at 0.05954 MeV energy. Thus, with the incorporation of 2.5 mole % of Bi_2_O_3_ to sample B1, the RPE values increase from 51.51% to 90.00%, indicating the sharp increase in shielding competency. The %RPE values are 8.77, 10.75, 11.60, 12.47, and 13.36 for the B1, A1, A2, A3, and A4 samples, respectively, at 2.506 MeV energy. Thus, the RPE values are at a maximum for A4 samples as they contain the highest mole% of Bi_2_O_3_. Moreover, the RPE values decrease with the increase in the energy, indicating that the shielding competency decreases with the increase in energy. At all the incident energies, RPE follows the trend A4 > A3 > A2 > A1 > B1. Thus, sample A4 has the best shielding properties among the fabricated samples.

**Figure 9:**
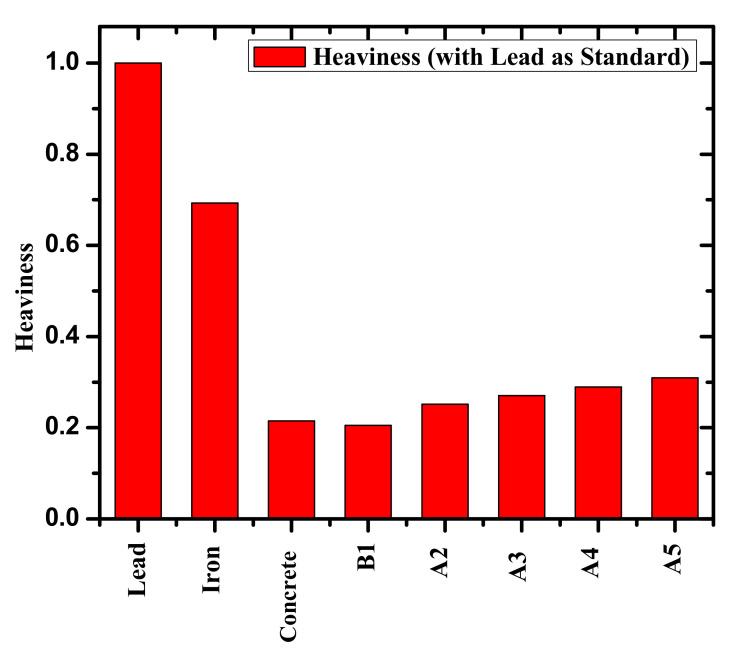
Comparison of heaviness of the present glasses with lead, iron, and concrete.

The investigation of the % heaviness of the fabricated glasses with different contents of Bi_2_O_3_ can be estimated by dividing the density of the glass by the density of the lead [20]. As given in Figure 9, B1 glass produced 20.5% of lead, while A1 glass produced 25.2% of lead and A4 glass produced 31.0% of lead. From Figure 9, we can note that the weight of iron (as an example of common shielding materials) is 69.3%. According to these results, the fabricated glasses can provide outstanding lightness even for the glass with a high content of Bi_2_O_3_ relative to traditional protection medium. From this parameter and the previous factors, the performance of the B1 and A1–A4 glasses as shielding materials is considerable, especially at higher contents of Bi_2_O_3_ and for the photons with low energy. Hence, the fabricated glasses have superiority to other traditional shielding materials (such as iron) in terms of lightness and transparency.

**Figure 10:**
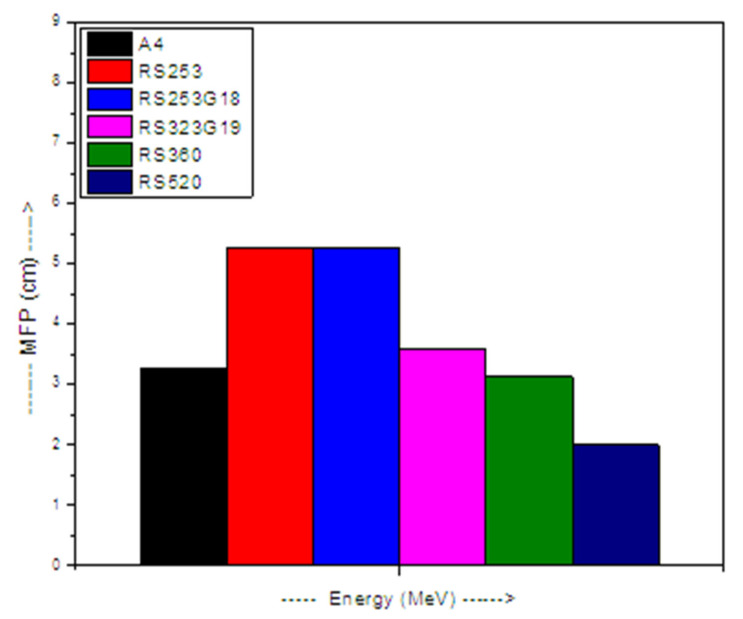
MFP comparison of glass A4 with commercial shielding glasses at 0.662 MeV.

The gamma ray shielding competence of the fabricated glasses was compared to that of the SCHOTT AG glasses by plotting the MFP of sample A4 (best shielding glass among the fabricated glasses) with some commercial shielding glasses at 0.662 MeV in Figure 10. The MFP of the glass A4 is 3.251 cm, whereas the MFPs of the RS253, RS253G18, RS323G19, RS360, and RS520 glasses are 5.263, 5.263, 3.571, 3.125, and 2 cm, respectively, at 0.662 MeV. Thus, we can conclude that the MFP of A4 glass is smaller as compared with the MFPs for the RS253, RS253G18, and RS323G19 glasses, whereas the MFP of A4 glass is higher as compared with the MFPs of the RS360 and RS520 glasses, respectively. Thus, the present A4 glass is a better shielding material compared with the RS253, RS253G18, and RS323G19 glasses.

## 4. Conclusions

The linear attenuation coefficient (LAC) for novel glass systems was experimentally calculated using NaI (Tl) scintillation detector and different radioactive point sources. The (MAC)_E_ and (MAC)_T_ are close together, with a small deviation reported (less than 4%). The compatibility between the practical and theoretical results shows the accuracy of the results obtained in the laboratory for determining the MAC of the prepared samples. The LAC results showed an increment in the attenuation ability with the addition of Bi_2_O_3_, thus at the seven examined energies, A4 possesses the highest LAC, owing to the highest amount of Bi_2_O_3_ present in this glass. Meanwhile, B1 glass, the glass with 0 mol% of Bi_2_O_3_, has the minimum LAC. Additionally, an upward trend in HVL was observed with the increasing energy from 0.0595 to 1.33 MeV. This result indicates that the increasing energy of thr photon causes an increase in the ability of the photons to transmit through the sample. This situation ascertains that more photons can penetrate the chosen glasses when the energy of the radiation increases. Moreover, an increasing trend in the Z_eff_ with the replacement of Na_2_O by Bi_2_O_3_ was reported. At any energy, A4 glass predominates over the remaining glasses owing to the high Bi_2_O_3_ content (10 mol%) in BTe4. It is also found that the influence of Bi_2_O_3_ on the Z_eff_ is significant at a low energy, and the Z_eff_ increases from 12.55 to 50.06 at 0.0595 MeV, from 11.71 to 41.21 at 0.081 MeV, and from 10.92 to 41.43 at 0.161 MeV. Meanwhile, at a higher energy (1.173 Mev, for example), Z_eff_ only increases from 10.73 to 14.75. The Z_ef_ follows the order B1 > A1 > A2 > A3 > A4, which suggested that increasing Bi_2_O_3_ has a positive effect on the photon shielding proficiencies.

## Figures and Tables

**Table 1:** The measured and theoretical MAC for the fabricated glasses.

Energy MeV	MAC (B1)		∆ (%)	MAC (A1)		∆ (%)	MAC (A2)		∆ (%)	MAC (A3)		∆ (%)	MAC (A4)		∆ (%)
	EXP	XCOM		EXP	XCOM		EXP	XCOM		EXP	XCOM		EXP	XCOM	
0.059	0.304	0.310	2.12	0.789	0.805	1.99	1.188	1.214	2.22	1.534	1.558	1.53	1.806	1.850	2.44
0.081	0.209	0.213	1.9	0.443	0.433	−2.3	0.603	0.614	1.88	0.750	0.767	2.21	0.883	0.897	1.52
0.161	0.140	0.138	−1.55	0.293	0.297	1.55	0.435	0.429	−1.25	0.531	0.540	1.66	0.613	0.635	3.52
0.356	0.097	0.099	2.3	0.115	0.119	2.9	0.136	0.135	−0.98	0.151	0.148	−1.75	0.163	0.160	−1.8
0.662	0.074	0.076	3.5	0.079	0.080	1.55	0.081	0.083	1.66	0.084	0.085	1.66	0.089	0.087	−1.46
1.173	0.057	0.058	0.88	0.059	0.058	−1.55	0.057	0.059	3.78	0.060	0.059	−1.22	0.058	0.059	2.52
1.332	0.055	0.054	−0.99	0.055	0.055	−0.98	0.055	0.055	−0.19	0.054	0.055	1.22	0.055	0.055	0.92

## Data Availability

The data presented in this study are available on request from the corresponding author.

## Abbreviations

MAC	mass attenuation coefficient
HVL	half value layer
Z_eff_	effective atomic number
I_0_	Initial intensity
I	transmitted intensity
f_i_	molar fraction of the ith constituent element
A_i_	atomic weight of the ith constituent element
Z_i_	atomic number of the ith constituent element
MFP	mean free path
RPE	radiation protection efficiency
